# Comparative evaluation of GSH, total protein and albumin levels in patients using smokeless tobacco with oral precancerous and cancerous lesions

**DOI:** 10.3892/mi.2024.139

**Published:** 2024-02-15

**Authors:** Anand Nimbal, Bhagyashri Ahirrao, Aruna Vishwakarma, Prashanth Vishwakarma, Alisha Bhushan Wani, Asmita Anant Patil

**Affiliations:** 1 Department of Dentistry, B M Patil Medical College, Hospital and Research Centre, Vijaypura, Karnataka 586103, India; 2 Department of Pathology, Jawahar Medical Foundation's ACPM Medical College, Dhule, Maharashtra 424001, India; 3 Department of Pedodontics, Jawahar Medical Foundation's ACPM Dental College, Dhule, Maharashtra 424001, India; 4 Department of Public Health Dentistry, Jawahar Medical Foundation's ACPM Dental College, Dhule, Maharashtra 424001, India; 5 Department of Conservative Dentistry and Endodontics, Jawahar Medical Foundation's ACPM Dental College, Dhule, Maharashtra 424001, India

**Keywords:** smokeless tobacco, serum albumin, reduced glutathione, serum protein, oral cancers

## Abstract

Smokeless tobacco (SLT) causes the excessive production of reactive oxygen species, leading to oxidative damage and carcinogenesis. The present study aimed to evaluate the levels of biomarkers, such as glutathione (GSH) in the blood, as well as serum albumin and total protein levels in SLT users with oral precancerous and cancerous lesions. A cross-sectional, prospective study was conducted on 240 patients aged 30-60 years, divided into four groups with 60 patients in each group as follows: Group 1, control group, non-tobacco users; group 2, 60 subjects with a history of SLT use and no oral lesions; group 3, SLT users with precancerous oral lesions; and group 4, SLT users with cancerous lesions. GSH levels in the blood, serum albumin levels and total protein levels were evaluated in all groups. ANOVA and Tukey's test post hoc were used to compare the levels of the biomarkers in all groups. Receiver operating characteristic curves were used to assess the reliability of the biomarkers, and regression analysis was used to determine the associations between the variables. The use of SLT was predominantly observed in males. The mean GSH and serum albumin levels were lowest in group 4 and highest in the control group (P<0.001). The total serum protein levels were higher in group 4 than in group 3. On the whole, as demonstrated herein, GSH and serum albumin were reliable biomarkers, whereas total protein was a weak biomarker. GSH and serum albumin levels may thus be efficiently used for the early diagnosis and prognosis of oral malignancies in SLT users

## Introduction

Smokeless tobacco (SLT) poses a significant public health concern in the Indian subcontinent, with India being widely regarded as the global epicenter of SLT usage (1). SLT, which does not involve combustion, is commonly employed intranasally or intraorally, predominantly in the form of snuffs or by chewing tobacco leaves (2). Specific varieties of SLT differ across various regions, resulting in a plethora of associated health risks. Presently, a multitude of SLT options are available, featuring diverse flavors and chewing habits, such as betel quid, khaini, mawa, pan masala plain and gutka (3). The prevalence and utilization of tobacco among young adults, specifically those aged ~30 years, are substantial. These demographics account for ~12% of global tobacco-related deaths, including the use of tobacco products and smokeless alternatives (4). Notably, the latter is considered to be a comparatively safe option for continuous cigarette smoking. It is crucial to recognize that tobacco is a significant risk factor for various chronic ailments, such as cancer, respiratory conditions and cardiovascular diseases (5).

The International Agency for Research on Cancer (IARC) has classified SLT as a group 1 carcinogen (6). Notably, tobacco-specific N-nitrosamines, namely 4-(methylnitrosamino)-1-(3-pyridyl)-1-butanone, nitrosonornicotine (NNN), N-nitrosoanatabine and N-nitrosoanabasine play a pivotal role in the production of free radicals (6). Concerning their corresponding oxidative stress actions, SLT extract is more harmful and results in oxidative tissue damage and apoptosis. Furthermore, it has been noted that the alkaline conditions present during betel nut chewing promote the generation of free radicals (7). At low or moderate levels, reactive oxygen species (ROS) and reactive nitrogen species (RNS) play crucial roles in the maturation of cellular structures and serve as tools for the host defense system. However, the excessive production of free radicals and oxidants leads to a phenomenon known as oxidative stress, which is a detrimental process capable of significantly modifying cell membranes and various structures, including proteins, lipids, lipoproteins and DNA (8). The administration of SLT at a low dose over a prolonged period of time can trigger oxidative stress, leading to detrimental effects on bodily tissues. These effects may play a role in the toxicity and carcinogenicity associated with the use of SLT (9).

Glutathione (GSH), an ubiquitous tripeptide thiol, is a vital intracellular and extracellular protective antioxidant. The intracellular and whole blood concentrations of GSH are in the millimolar range, whereas the plasma concentration is in the micromolar range accounting for ~0.4% of the total blood GSH levels (10). Of note, GSH is detected at high concentrations (5 mM) in the majority of cells. It plays a crucial role in shielding cellular macromolecules from endogenous and exogenous ROS and RNS. GSH directly scavenges diverse oxidants, such as superoxide anions, hydroxyl radicals, nitric oxide and carbon radicals (11). Studies have indicated that antioxidants exert their safeguarding effects by reducing oxidative DNA impairment and inhibiting the initiation and progression of carcinogenesis (12,13). This notion serves as a significant gauge for evaluating the mechanisms of antioxidant defense in the context of malignancy.

In a blood sample with a pH of 7.4, ~69% of nicotine exists in an ionized state, 31% remains unionized and <5% attaches to plasma proteins (14). The occurrence of hypoalbuminemia in patients with oral cancer may be attributed to the effects of free radical-mediated protein oxidation and the subsequent reduction in the protective antioxidant defense mechanism (15). The levels of oxidized proteins in plasma are noteworthy indicators of oxidative stress originating from free radicals. Protein oxidation plays a crucial role in the pathogenesis of oral cancers. Hyperproteinemia, which manifests as cachexia, is a common occurrence in oral malignancies. Therefore, serum proteins could potentially function as pivotal diagnostic and prognostic markers for oral premalignant diseases and oral malignancies. Researchers have examined the associations between enzymes, proteins and glycoproteins, and have found significant changes in the levels of protein biomarkers in blood serum (16). Additionally, SLT use leads to a significant decrease in serum albumin levels and alterations in liver enzyme levels. This is likely due to the damage and destruction of the liver tissue caused by the components of SLT, which has been proven to activate microsomal enzymes in liver cells (2). Therefore, the aim of the present study was to evaluate and compare the levels of blood GSH, total plasma protein and albumin in SLT users with precancerous and cancerous lesions, compared to non-tobacco users.

## Patients and methods

The present cross-sectional observational study was conducted at the Department of Public Health Dentistry, Jawahar Medical Foundation's ACPM Dental College (Dhule, India) between January, 2022 and December, 2022. Approval was obtained from the Institutional Ethics Committee of Jawahar Medical Foundation Annasaheb Chudaman Patil Memorial Dental College Dhule (EC/NEW/INST/2022/2959/Y22/212). Written informed consent was obtained from all participants after the study protocol was explained to them. The present study was conducted in accordance with the principles of the Declaration of Helsinki and followed the Strengthening the Reporting of Observational studies (STROBE) guidelines.

### Sample size calculation

The sample size was calculated using G*Power software (latest ver. 3.1.9.7; Heinrich-Heine-Universität Düsseldorf, Düsseldorf, Germany). The standard deviation was taken from a previous study, to detect the effect size of 0.5 mg/dl in of GSH in the oral cancer and control groups (17). The power of the study was 80%, with a 95% confidence interval, and type 1 error of 5%. The sample size was calculated as 60 patients per group.

### Selection criteria

**Figure 1:**
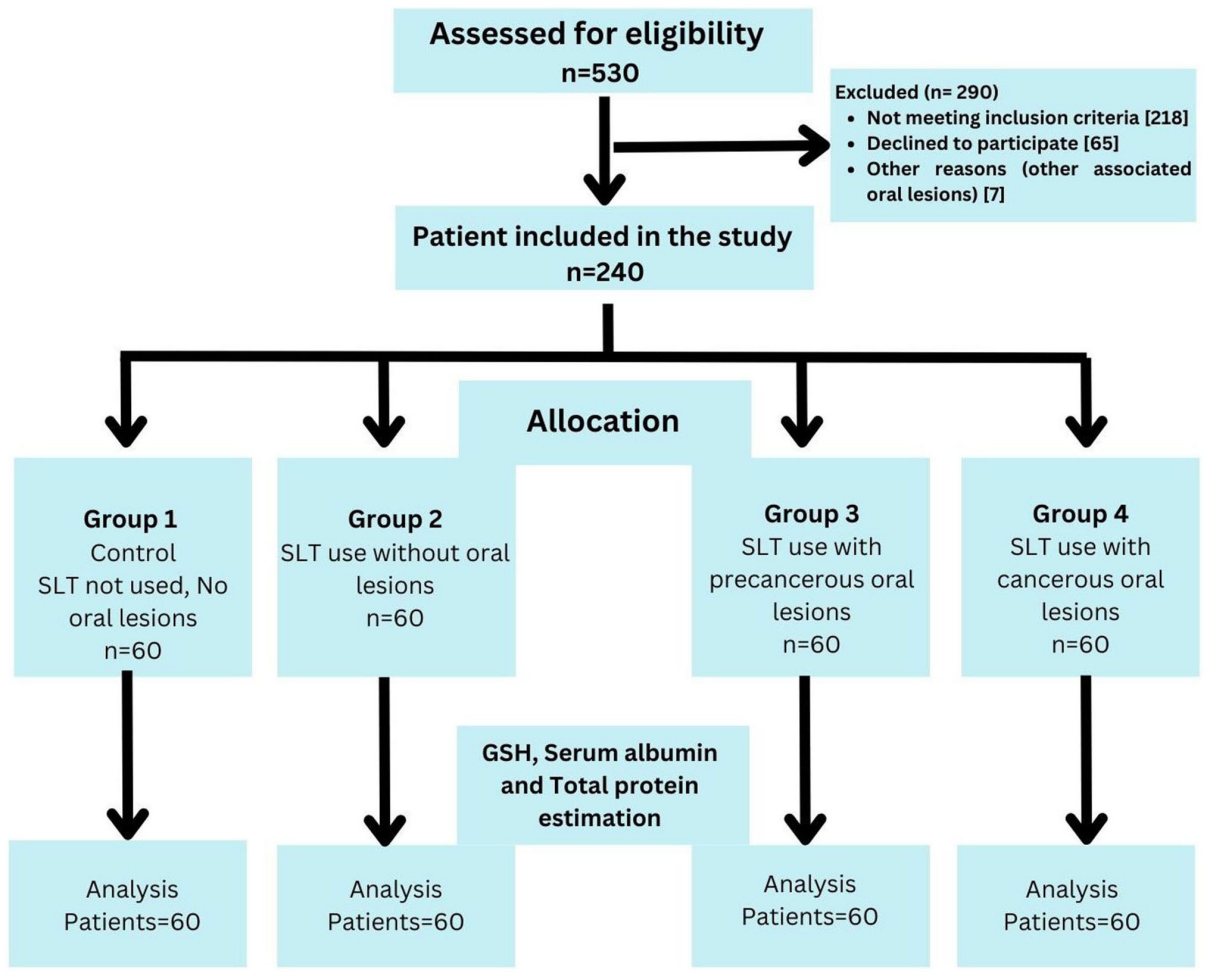
Strengthening the Reporting of Observational studies (STROBE) flow diagram of the process used in the present study for patient selection.

A total of 1,500 patients were screened in cancer awareness camps in the district of Dhule to select a sample of 240 patients aged 30-60 years, based on the selection criteria of the study. In total, 240 patients were divided into four groups as follows: Group 1 (control group) comprised 60 healthy individuals with no history of SLT use; group 2 comprised 60 healthy individuals with at least a 1-year history of SLT use, but without the occurrence of any oral precancerous or cancerous lesions; group 3 comprised 60 clinically and histopathologically confirmed individuals with oral precancerous lesions who had not received any prior treatment with at least a 1-year history of SLT use; and group 4 comprised 60 clinically and histopathologically confirmed individuals with oral squamous cell carcinoma without metastasis, who had not received any prior treatment with at least a year history of SLT use. Individuals >30 years of age who were willing to participate in the study and had provided written informed consent were included in the study. Individuals with any systemic disease, cancer patients undergoing treatment or radiotherapy, pregnant or lactating females, individuals using tobacco smoking, those taking any medications for >3 months, those with a previous history of malignancy or a history of antioxidant medication, and those taking corticosteroids over the past 6 months were excluded from the study. A flow diagram of the study groups and selection process is presented in Fig. 1.

### Procedure

The participants were instructed to fast overnight. The following day, between 7 and 9 a.m., a total of 5 ml blood was drawn from the mid-cubital vein with the necessary aseptic precautions in a 5-ml disposable syringe and transferred to sterile tubes containing ethylenediaminetetraacetic acid (EDTA) to prevent coagulation. The blood was centrifuged at 1,007 x g for 7 min and at a temperature of 8 to 12˚C in the centrifuge. The plasma and buffy coat were separated, and plasma was used for the estimation of glutathione, whereas serum was used for the estimation of total protein and albumin. GSH, total protein and albumin levels were estimated by a trained pathologist (the author BA) at the Pathology Laboratory of ACPM Medical College, Dhule, India. Routine blood investigations were performed in all patients.

### Estimation of GSH

The GSH levels were estimated using the method described in the study by Beutler *et al* (18), which is a simple and accurate method for determining GSH levels in blood. This method is based on the development of a relatively stable yellow color produced by the reaction of Ellman's reagent [5,5'-dithiobis-(2-nitrobenzoic acid) or DTNB reagent (MerckMillipore)] with GSH to form TNB chromophore, which has a maximum absorbance at 412 nm. The rate of TNB formation, measured at 412 nm, was proportional to the concentration of GSH in the sample. The rate of change in absorbance (∆A412 nm/min) was linear for the convenience and consistency of the measurement, and was linearly proportional to the total concentration of GSH. The optical density of the solution was measured at 412 nm using a spectrophotometer (Manti Lab Solutions), and the value of GSH was computed as mg/g Hb (18).

### Estimation of serum protein levels

The serum protein levels were estimated using the Biuret method (19). Biuret reagent (MerckMillipore) comprised of sodium hydroxide and hydrated copper sulfate, together with potassium sodium tartrate, the latter of which was added to chelate and thus stabilized the cupric ions. The reaction of the cupric ions with the nitrogen atoms involved in peptide bonds led to displacement of the peptide hydrogen atoms under alkaline conditions. A tri- or tetra-dentate chelation with the peptide nitrogen produced a characteristic violet color. The intensity of the color, which had a maximum absorption at 540 nm using a spectrophotometer (Manti Lab Solutions), was proportional to the protein concentration.

### Estimation of serum albumin levels

The serum albumin levels were estimated using the spectrophotometric method described by Rodkey (20). In this method, the serum was diluted with a solution of bromocresol green (MerckMillipore) at a sufficient concentration to allow an essentially linear change in absorbance with the albumin concentration. Measurements were made at 615 nm with spectrophotometer (Manti Lab Solutions), where the absorbance of hemoglobin or bilirubin did not interfere.

### Statistical analysis

Data were analyzed using SPSS software version 22 (IBM Corp.). The Shapiro-Wilk test was used to assess the normal distribution of the data. As the data were normally distributed, parametric tests, such as one-way analysis of variance (ANOVA), followed by post hoc analysis with Tukey's test, were used to assess the differences between the groups. For ordinal data, the Chi-squared test and Fisher's exact test were used. Regression analysis was used to assess the associations between variables, and receiver operating curves (ROC) were used to evaluate biomarker performance. A value of P≤0.05 was considered to indicate a statistically significant difference.

## Results

### Descriptive analysis

The descriptive analysis in the present study did not reveal any significant differences in the mean age of the patients in the different the groups (P>0.05), whereas statistically significant differences were observed in the duration of SLT use between groups 2, 3 and 4. The maximum duration of SLT use was 14.15±5.81 years, observed in SLT users who developed oral cancer (group 4), whereas the minimum duration of SLT use was 6.15±2.35 years, observed in SLT users with precancerous oral lesions. All groups had non-significant distributions of males and females, although the habit of SLT use was more prevalent in males than in females. Gutka chewing was the most common form of SLT, followed by betel quid chewing. The frequency of SLT chewing/per day was the highest in group 4, followed by groups 3 and 2. The majority of the participants in group 2 chewed SLT for <5 min (70%), whereas the majority of the participants in group 4 chewed SLT for >5 min (63%), as depicted in Table I. Post hoc analysis revealed that there were statistically notable disparities in the duration of SLT use between groups 2 and 3 in comparison to group 4. Nevertheless, there were no significant differences between groups 2 and 3. Statistically significant disparities were observed among all experimental groups in terms of the frequency of SLT use (Table II).

The most common precancerous lesion in group 3 was leukoplakia (60%), followed by oral submucosal fibrosis (23%) and erythroplakia (17%). The majority of the participants in group 4 had well-differentiated oral squamous cell carcinoma (52%) (Table III). The majority of oral lesions were present on the buccal mucosa, labial mucosa and gingivobuccal corridor.

### Hematological analysis

Statistically significant differences were observed in all hematological parameters between the groups. Group 4 exhibited a significant decrease in mean hemoglobin levels, total platelet count and total leukocyte count, followed by groups 3 and 2 compared to the control group. The total red blood cell (RBC) count, erythrocyte sedimentation rate, mean corpuscular hemoglobin and high-sensitivity C-reactive protein (hs-CRP) levels were significantly higher in group 4, followed by groups 3 and 2, compared with the control group (Table IV). Post hoc analysis revealed significant differences between groups 1 and 4 in terms of Hb levels. Moreover, there were significant differences between all groups, except for groups 3 and 4, in terms of the total RBC count. Additionally, significant differences were found between groups 1 and 3, groups 1 and 4, and groups 2 and 4 in terms of total platelet count. Furthermore, there were significant differences between all groups, except groups 2 and 3, as well as groups 2 and 4, in terms of the total leukocyte count. Moreover, significant differences were found between all groups, except for groups 2 and 3, as well as groups 3 and 4, in terms of MCH. Lastly, there were significant differences between all groups, except for Groups 1 and 2, in terms of hs-CRP levels (Table IV).

### Inferential analysis

Analysis between the groups using ANOVA revealed statistically significant differences in the mean values of GSH, serum protein and albumin levels (P<0.001). The maximum levels of reduced GSH (9.61±0.86 mg/Hb) were observed in healthy individuals with no history of SLT use (group 1), and the minimum levels (2.63±0.43) were found in group 4, as shown in Table V. Post hoc analysis using Tukey's test revealed a statistically significant difference in serum GSH levels between all groups (P<0.001; Table VI). No significant differences were observed in the serum protein levels between groups 1 and 2, groups 1 and 4, and groups 2 and 4, whereas no significant differences were observed in the serum albumin levels between groups 2 and 3, as shown in Table VI.

### Results of ROC analysis

**Figure 2:**
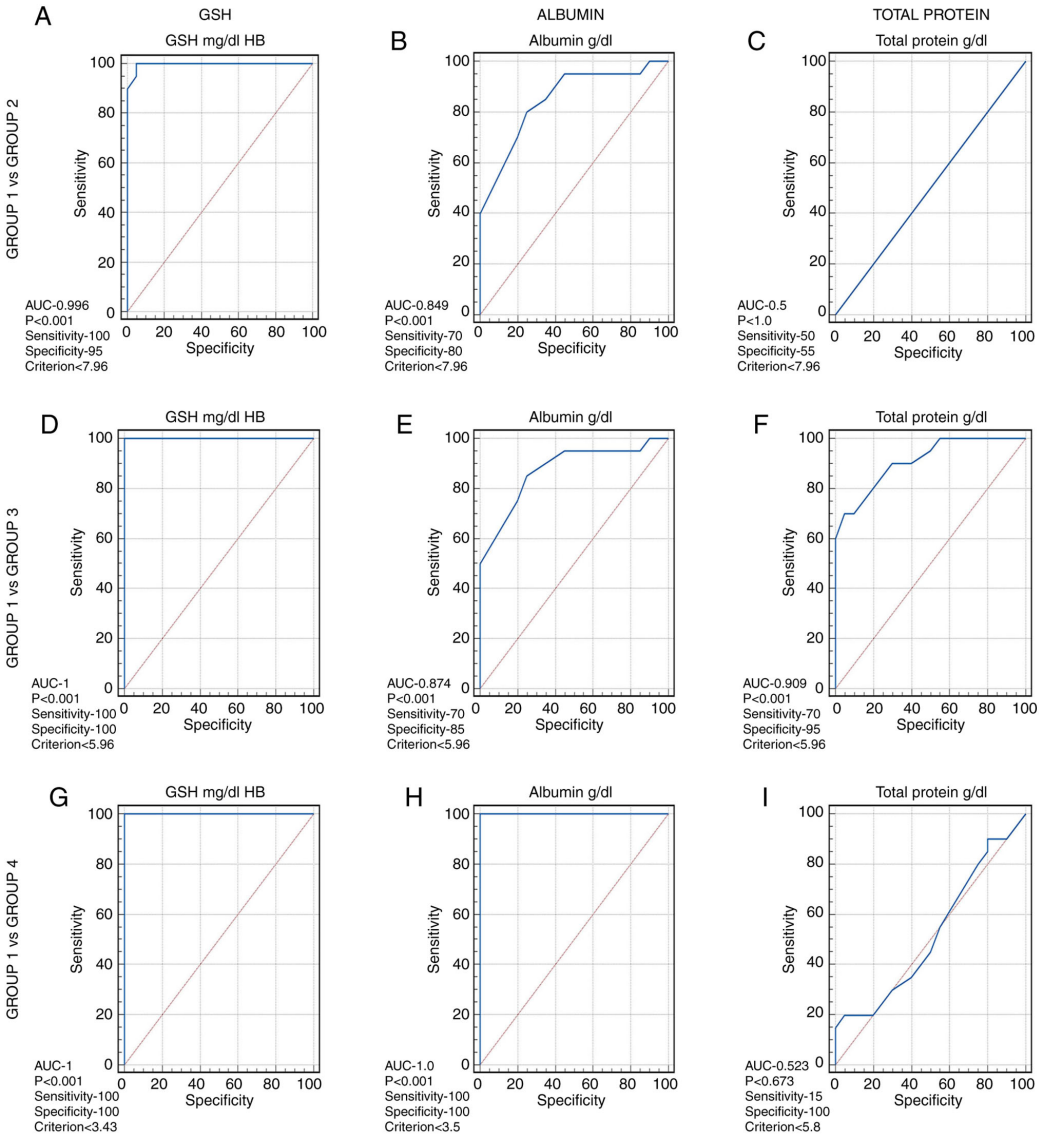
Receiver operating characteristics analysis. (A-C) Group 1 vs. group 2 for GSH, albumin and total protein levels. (D-F) Group 1 vs. group 3 for GSH, albumin and total protein levels. (G-I) Group 1 vs. group 4 for GSH, albumin and total protein levels. GSH, glutathione; AUC, area under the curve.

ROC analysis was conducted to determine the sensitivity and specificity of GSH in the blood and albumin and total protein levels in the serum at different thresholds. The optimum threshold values of GSH was found to be ≤7.96 for group 2 (Fig. 2A), ≤5.96 for group 3 (Fig. 2D) and ≤3.43 for group 4 (Fig. 2G), and having a sensitivity and specificity of 100%. Albumin had a threshold value of ≤7.9 for group 2 with 70% sensitivity and 80% specificity (Fig. 2B), ≤5.9 for group 3 with 70% sensitivity and 85% specificity (Fig. 2E), and ≤3.5 for group 4 (Fig. 2H), with 100% sensitivity and 100% specificity. The threshold values of total serum protein levels were >7.9 for group 2 (Fig. 2C) at 50% sensitivity and 55% specificity, ≤5.96 for group 3 (Fig. 2F) at 70% sensitivity and specificity of 95%, and ≤5.81, at 15% sensitivity and 100% specificity for group 4 (Fig. 2I). This indicates that GSH and albumin levels are reliable diagnostic biomarkers, whereas the total protein content is a weak biomarker.

### Regression analysis

**Figure 3:**
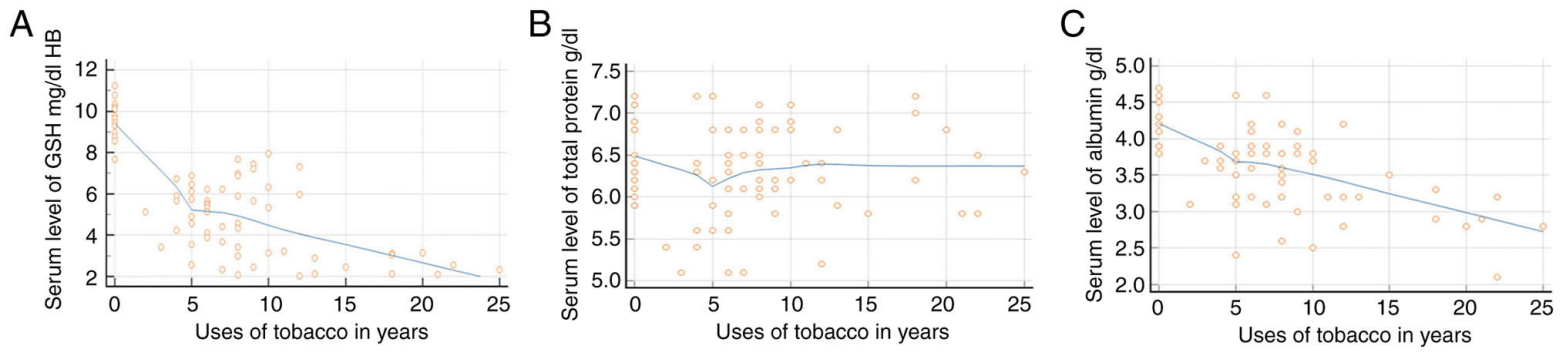
Regression analysis illustrating the association of duration of smokeless tobacco usage and the levels of (A) GSH, (B) total protein and (C) albumin. GSH, glutathione.

**Figure 4:**
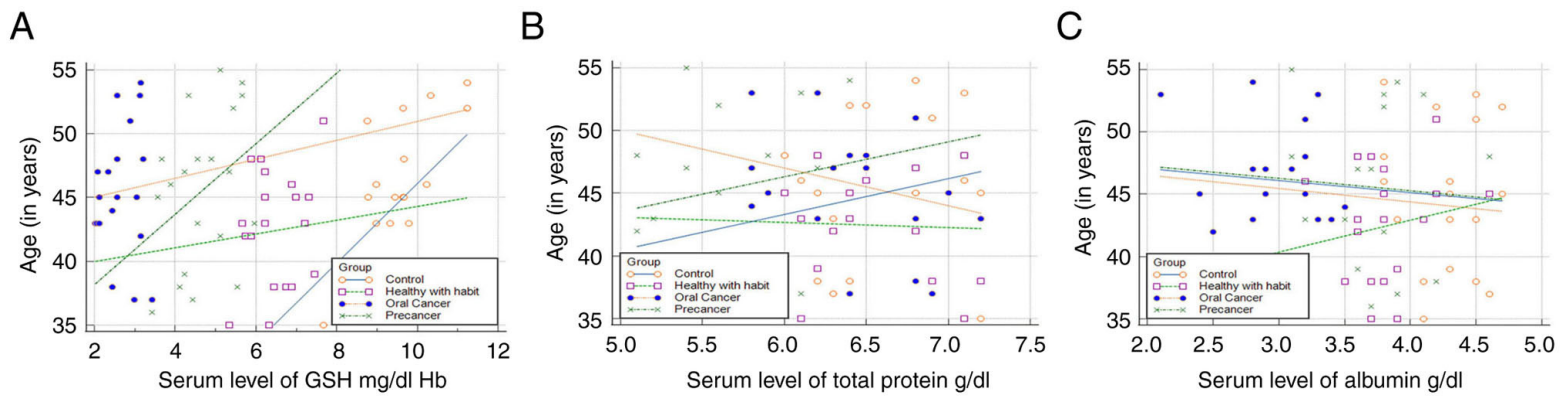
Regression analysis illustrating association between age and the levels of (A) GSH, (B) total protein and (C) albumin. GSH, glutathione.

Regression analysis revealed that the GSH levels in the blood and serum albumin levels decreased as the duration of SLT use increased; however, the total protein level only exhibited slight fluctuations in the initial phases of tobacco use (Fig. 3). The GSH levels exhibited an increasing trend with age, indicating that the GSH levels increased in all groups as the age of the patients increased. The total protein level decreased in group 4, whereas groups 3 exhibited an increasing trend with age. The serum albumin levels exhibited an increasing trend with age only in group 2 (Fig. 4).

## Discussion

In healthy humans, a state of equilibrium is maintained between oxidants and antioxidants. Nevertheless, in an atypical state, an over-abundance of oxidizing agents is generated, leading to the inhibition of antioxidant defenses. Consequently, this results in a deviation in the ratio favoring pro-oxidants (8). SLT alters the activity of antioxidants in saliva. Oxidative stress can function as a biomarker for the diagnosis and prediction of damage and the abnormal growth of oral tissues. Additionally, it can serve as an early indicator to prevent abnormal changes in the oral cavity (9).

The present study found the increased use of SLT in males aged 45 years, which was in agreement with the findings of previous studies (1,2,4,7). There is substantial evidence to indicate that the enactment of smoke-free laws, the augmentation of smoking taxes and the prevalence of a favorable societal attitude towards SLT during working hours have been positively associated with the rate of SLT consumption among males (4). Substances such as lime and catechu, which are employed in the formulation of SLT commodities, participate in the generation of ROS within the cellular environment (14).

In the present study, GSH levels were higher in non-tobacco users than in tobacco users. Tobacco snuffs can generate free radicals, causing protein nitration, lipid peroxidation and DNA adduct formation. Exposure to SLT leads to the generation of ROS, which are considerably higher in SLT users than in non-tobacco users (12,13). GSH, a vital antioxidant that dissolves in water, is produced through the synthesis of the amino acids, glycine, glutamate and cysteine. GSH exhibits an elevated redox potential and effectively operates as a potent antioxidant (10). It facilitates the detoxification and breakdown of ROS, thereby inhibiting cellular oxidative damage and cancer development.

Herein, GSH levels were found to be decreased in SLT users with precancerous and cancerous lesions. The lowest GSH levels were observed in SLT users with oral cancer. The findings of the present study are consistent with those of previous studies (5,17,21,22). Glutathione consists of a reduced form known as GSH and a form that has been oxidized, referred to as GSH disulfide (GSSG). The ability of GSH to be regenerated is directly related to the redox state of the GSSG-GSH couple (GSSG/2GSH). As a result, GSH provides primary support for intracellular ‘redox homeostasis’ or ‘redox buffering’ capacity (22). When subjected to excessive xenobiotics, including carcinogens, there is an increased utilization of GSH for conjugation, which in turn leads to detoxification. This process results in a decrease in the GSH/GSSG ratio, rendering GSH less available, and consequently reducing the body's defense against free radicals. Notably, the depletion of GSH is adequate to sensitize cancer cells to both oxidative and nitrative stress. This sensitization leads to DNA damage. Consequently, DNA degeneration can occur, which can activate carcinogens and ultimately initiate and progress into cancer. Depletion of GSH may sensitize tumors to chemotherapy and radiotherapy (10,16). ROC analysis revealed GSH to be a reliable biomarker for predicting oral cancer. In the present study, the mean GSH levels decreased with age and increased with the duration of SLT use, which is in agreement with the findings of previous studies (23,24).

Serum proteins have been widely acknowledged for their antioxidant characteristics owing to the presence of unbound thiol groups. Of all the proteins, albumin stands out as the most efficacious and abundant extracellular antioxidant. In the present study, it was found to be a reliable biomarker. The findings of the present study indicated a significant decrease in serum albumin levels in SLT users compared to healthy controls, and it decreased further in patients with precancerous and cancerous lesions. This finding is in accordance with that of previous studies (9,15,25). The effect of free radicals formed by SLT has been demonstrated to induce modifications to DNA bases, causing fractures in the DNA strand, impairing the integrity of tumor suppressor genes and promoting the expression of proto-oncogenes. Additionally, these free radicals can disrupt antioxidant defense mechanisms. Albumin is considered to possess the ability to function as an antioxidant, which may be attributed to the abundant presence of sulfhydryl and free thiol groups within the molecular structure of albumin. Oral cancers are associated with inflammatory mediators, such as IL-6 and TNF, which potentially exert their effects through dual mechanisms, namely, by enhancing the extravasation of albumin across the capillary endothelium at the tumor site and dampening the hepatic production of albumin. A decrease in serum albumin levels can lead to a reduction in the sequestration of unbound reactive molecules, thereby enhancing the probability of deleterious cellular damage that can induce cancer development (26).

In the present study, serum protein levels also decreased with the use of SLT and during the precancerous phase. However, there was no significant difference in serum protein levels between the control group and SLT users with oral cancer. This finding is in agreement with the findings of previous studies (13,27). The decrease in the serum protein concentration can potentially be elucidated in relation to the inflammatory response associated with oral malignancies. The total serum protein level was found to be a weak biomarker in the present study.

The findings of the present study revealed that reduced glutathione and serum albumin are reliable biomarkers for the early diagnosis and the assessment of the prognosis of oral premalignant and malignant lesions, and for planning specific treatment strategies for their prevention and management. These findings are of particular importance for subjects who have the habit of using SLT. They should be encouraged to quit these habits, and awareness campaigns should be organized where these biomarkers can be used efficiently for mass screening.

The altered hematological parameters in SLT users suggest the selective toxicity of SLT and its components. The increase in the RBC count in SLT users observed herein indicated erythropoiesis, which may be due to insufficient pulmonary function with the long-term use of SLT. Elevated hs-CRP levels also suggest the presence of chronic inflammation and an increased risk of cardiovascular disease in SLT users (28).

A limitation of the present study was the evaluation of GSH, albumin and serum proteins in the blood samples of patients, but not in salivary samples. The primary challenge hindering the development of a salivary diagnostic protocol lies in the fact that although numerous biomarkers identified in the blood serum can also be detected in saliva, their concentrations are so minimal that they offer limited value to the diagnostic process. Moreover, saliva collection is highly sensitive and affects the salivary biomarker levels. The present study also did not consider variations in biomarker levels at the different stages of oral squamous cell carcinoma. The present study evaluated GSH levels in the blood samples of patients; however, the estimation of GSH levels in blood and tissue samples of patients could have provided more information on oxidative stress. As GSH levels have been associated with oxidative stress (29), future studies should be conducted to assess ROS, and enzymes such as glutathione-peroxidase, and catalase at the cellular level. The present study measured the total serum protein levels, which were found to be weak biomarkers. Therefore, future prospective studies need to be conducted to evaluate specific proteins in salivary or serum samples from patients at different stages of oral cancer.

In conclusion, the findings of the present study draw a conclusion regarding the impact of oral premalignant and malignant tumors, on the concentration of GSH and proteins in the sera in comparison with individuals without any health issues or tobacco habits, indicating the possibility of utilizing these changes in GSH and protein profiles to diagnose and predict the course of oral cancer.

## Figures and Tables

**Table I:** Demographic details of the study participants.

Variable	Group 1 (Control, no SLT)	Group 2 (SLT use, no lesions)	Group 3 (SLT use, precancerous lesions)	Group 4 (SLT use, cancerous lesions)	P-value
Age, years (mean ± SD)^a^	44.9±5.67	44.4±4.27	45.6±5.84	45.5±5.87	0.595 (NS)
Sex, n (%)^b^					
Male	39 (65%)	40 (66%)	42 (70%)	43 (71%)	0.856 (NS)
Female	21 (35%)	20 (34%)	18 (30%)	17 (29%)	
Type of SLT use, n (%)^c^					
Betel quid	NA	18 (30%)	12 (20%)	20 (33%)	0.7710 (NS)
Gutka	NA	30 (50%)	36 (60%)	32 (53%)	
Mawa	NA	5 (9%)	4 (6%)	2 (4%)	
Naswar	NA	4 (6%)	6 (10%)	4 (6%)	
Other	NA	3 (5%)	2 (4%)	2 (4%)	
Duration of SLT use, years (mean ± SD)^a^	NA	7.05±2.15	6.15±2.35	14.51±5.81	0.0001^d^
Frequency of SLT use (per day) (mean ± SD)^a^	NA	5.78±3.32	7.38±2.12	8.96±2.08	0.0001^d^
Retention of SLT, n (%)^b^					
<5 min	NA	42 (70%)	28 (47%)	22 (37%)	0.009^d^
>5 min	NA	18 (30%)	32 (53%)	38 (63%)	

Data were analyzed using

^a^one-way ANOVA,

^b^the Chi-squared test, or

^c^Fisher's exact test;

^d^statistically significant differences (P<0.05). SD, standard deviation; SLT, smokeless tobacco; NA, not applicable; NS, not significant (P>0.05).

**Table II:** Post hoc analysis of the duration and frequency of SLT use in groups 2, 3 and 4.

Parameter and pairwise comparison	P-value
Duration of SLT use (years; mean ± SD)	
Group 2 vs. group 3	0.403 (NS)
Group 2 vs. group 4	0.001^a^
Group 3 vs. group 4	0.001^a^
Frequency of SLT use (per day; mean ± SD)	
Group2 vs. group 3	0.002^a^
Group 2 vs. group 4	0.002^a^
Group 3 vs. group 4	0.002^a^

The groups were as follows: Group 1, control (no SLT); group 2, SLT use (no lesions); group 3, SLT use (precancerous lesions); group 4, SLT use (cancerous lesions).

^a^Statistically significant differences (P<0.05). SD, standard deviation; SLT, smokeless tobacco; NS, not significant (P>0.05).

**Table III:** Histopathological details of lesions in groups 3 and 4.

Group	Histopathological grade			
Group 3 (SLT use, precancerous lesions; n=60)	Mild dysplasia	Moderate dysplasia	Severe dysplasia	Carcinoma *in situ*
Leukoplakia (n=36, 60%)	n=24 (66%, 40%)	n=8 (23%, 12%)	n=3 (8%, 5%)	n=1 (3%, 2%)
Erythroplakia (n=10, 17%)	n=3 (0.3%, 5%)	n=4 (0.4%, 7%)	n=2 (0.2%, 3%)	n=1 (0.1%, 2%)
Oral submucous fibrosis (n=14, 23%)	n=9 (64%, 15%)	n=4 (29%, 7%)	n=1 (7%, 2%)	n=0 (0%)
	Well-differentiated	Moderately differentiated	Poorly differentiated	Metastasis
Group 4 (SLT use, cancerous lesions; n=60)				
Squamous cell carcinoma (n=60)	32 (52%)	20 (34%)	8 (14%)	0 (0%)

In the parentheses in which two different percentages are listed, the percentage on the left was obtained out of the patients in each sub-category, whereas the one on the right was obtained out of the patients in the group. SLT, smokeless tobacco.

**Table IV:** Comparison of hematological investigations of the study groups using one-way ANOVA followed by post hoc analysis.

Parameter	Group	Mean ± SD	P-value	Pairwise comparison	P-value
Hb%	Group 1	14.20±3.02	0.015^a^	Group 1 vs. group 2	0.740
	Group 2	13.74±1.98		Group 1 vs. group 3	0.130
	Group 3	13.20±1.94		Group 1 vs. group 4	0.010^a^
	Group 4	12.80±2.92		Group 2 vs. group 3	0.640
				Group 2 vs. group 4	0.170
				Group 3 vs. group 4	0.820
Total RBCs (10^6^/µl)	Group 1	4.81±0.02	0.001^a^	Group 1 vs. group 2	0.001^a^
	Group 2	5.14±0.57		Group 1 vs. group 3	0.001^a^
	Group 3	5.67±0.62		Group 1 vs. group 4	0.001^a^
	Group 4	5.82±0.32		Group 2 vs. group 3	0.001^a^
				Group 2 vs. group 4	0.001^a^
				Group 3 vs. group 4	0.265
ESR (mm/h)	Group 1	13.24±6.24	0.183	Group 1 vs. group 2	0.320
	Group 2	15.43±7.21		Group 1 vs. group 3	0.540
	Group 3	14.95±6.96		Group 1 vs. group 4	0.160
	Group 4	15.91±7.76		Group 2 vs. group 3	0.980
				Group 2 vs. group 4	0.980
				Group 3 vs. group 4	0.870
Total platelets (lakhs/mm^3^)	Group 1	3.24±1.21	0.001^a^	Group 1 vs. group 2	0.801
	Group 2	2.91±2.14		Group 1 vs. group 3	0.001^a^
	Group 3	1.98±2.32		Group 1 vs. group 4	0.001^a^
	Group 4	1.89±2.12		Group 2 vs. group 3	0.054
				Group 2 vs. group 4	0.028^a^
				Group 3 vs. group 4	0.994
TLC (10^3^/mm^3^)	Group 1	9.23±1.91	0.001^a^	Group 1 vs. group 2	0.007
	Group 2	8.24±1.22		Group 1 vs. group 3	0.001^a^
	Group 3	7.91±1.61		Group 1 vs. group 4	0.001^a^
	Group 4	7.23±1.84		Group 2 vs. group 3	0.699
				Group 2 vs. group 4	0.005^a^
				Group 3 vs. group 4	0.117
MCH (pg)	Group 1	29.62±1.92	0.001^a^	Group 1 vs. group 2	0.001^a^
	Group 2	32.58±1.41		Group 1 vs. group 3	0.001^a^
	Group 3	33.41±1.86		Group 1 vs. group 4	0.001^a^
	Group 4	34.06±1.84		Group 2 vs. group 3	0.052
				Group 2 vs. group 4	0.001^a^
				Group 3 vs. group 4	0.186
hs-CRP (mg/l)	Group 1	2.56±1.53	0.001^a^	Group 1 vs. group 2	0.238
	Group 2	3.63±1.98		Group 1 vs. group 3	0.001^a^
	Group 3	12.21±3.43		Group 1 vs. group 4	0.001^a^
	Group 4	16.89±4.56		Group 2 vs. group 3	0.001^a^
				Group 2 vs. group 4	0.001^a^
				Group 3 vs. group 4	0.001^a^

The groups were as follows: Group 1, control (no SLT); group 2, SLT use (no lesions); group 3, SLT use (precancerous lesions); group 4, SLT use (cancerous lesions).

^a^Statistically significant differences (P<0.05). SD, standard deviation; SLT, smokeless tobacco; Hb, hemoglobin; RBC, red blood cells; ESR, erythrocyte sedimentation rate; TLC, total leukocyte count; MCH, mean corpuscular hemoglobin; hs-CRP, high-sensitivity C-reactive protein.

**Table V:** Comparative analysis of the different variables using ANOVA.

Group	Average level of GSH in blood, mg/Hb (mean ± SD)	Average serum level of total protein, g/dl (mean ± SD)	Average serum level of albumin, g/dl (mean ± SD)
Group 1 (n=60)	9.61±0.86	6.56±0.41	4.25±0.29
Group 2 (n=60)	6.56±0.70	6.46±0.34	3.81±0.28
Group 3 (n=60)	4.68±0.74	5.74±0.42	3.68±0.37
Group 4 (n=60)	2.63±0.43	6.51±0.44	2.97±0.35
Value from ANOVA	0.001^a^	0.001^a^	0.001^a^

^a^P<0.05, indicates a statistically significant difference. The groups were as follows: Group 1 (control), no SLT use; group 2, SLT use, no lesions; group 3, SLT use, precancerous lesions; group 4, SLT use, cancerous lesions. SLT, smokeless tobacco; GSH, glutathione.

**Table VI:** Intra-group comparisons of different variables using Tukey's post-hoc test.

Groups	Average serum level of GSH mg/Hb	Average serum level of total protein g/dl	Average serum level of albumin g/dl
Group 1 vs. group 2	0.001^a^	0.595 (NS)	0.001^a^
Group 1 vs. group 3	0.001^a^	0.001^a^	0.001^a^
Group 1 vs. group 4	0.001^a^	0.908 (NS)	0.001^a^
Group 2 vs. group 3	0.001^a^	0.001^a^	0.124 (NS)
Group 2 vs. group 4	0.001^a^	0.938 (NS)	0.001^a^
Group 3 vs. group 4	0.001^a^	0.001^a^	0.001^a^

^a^P<0.05, indicates a statistically significant difference. The groups were as follows: Group 1 (control), no SLT use; group 2, SLT use, no lesions; group 3, SLT use, precancerous lesions; group 4, SLT use, cancerous lesions. SLT, smokeless tobacco; GSH, glutathione.

## Data Availability

The datasets used and/or analyzed during the current study are available from the corresponding author on reasonable request.
